# Highly Electroactive Frozen-State Polymerized Polypyrrole Nanostructures for Flexible Supercapacitors

**DOI:** 10.3390/polym15204140

**Published:** 2023-10-18

**Authors:** Doebner Von Tumacder, Islam M. Minisy, Oumayma Taboubi, Patrycja Bober

**Affiliations:** 1 Institute of Macromolecular Chemistry, Czech Academy of Sciences, 162 00 Prague, Czech Republic; tumacder@imc.cas.cz (D.V.T.); minisy@imc.cas.cz (I.M.M.); taboubi@imc.cas.cz (O.T.); 2 Faculty of Science, Charles University, 128 43 Prague, Czech Republic

**Keywords:** polypyrrole, frozen-state polymerization, methyl orange, Acid Blue 25, supercapacitors

## Abstract

The polymerization of pyrrole in the frozen state with the presence of organic dyes (methyl orange (MO) and Acid Blue 25 (AB)) has proven to produce polypyrrole (PPy) nanostructures. Herein, we explore the electrochemical properties of PPy prepared under frozen-state conditions (−24 °C) with and without the presence of organic dyes. The electroactivity of PPy prepared with MO and AB significantly increased in all electrolytic media with a capacitance higher than this of the PPy prepared at room temperature. The highest capacitance (1914 F g^−1^) was obtained for PPy-MO in 0.2 M HCl solution. The impedance spectra of PPy showed a decrease in charge transfer resistance when the dyes were present. This indicates a conductivity increase of PPy. Improved electrochemical stability was observed for PPy, PPy-MO, and PPy-AB prepared at −24 °C, wherein a steady gain of capacitance was maintained during 5000 potential cycling. In addition, a PPy-based supercapacitor device was fabricated to demonstrate the energy storage characteristics of PPy, where it showed good capacitive behavior and stability. Overall, frozen-state polymerized PPy posed an impressive capacitive performance for flexible supercapacitors.

## 1. Introduction

Increasing attention to the development of flexible supercapacitors is on high demand for energy storage devices due to their structure, good flexibility, light weight, and outstanding electrochemical performance [1,2,3,4]. Supercapacitors in general possess good energy storage abilities, and at the same time, they can provide high power output. In this regard, the utilization of polymers as supercapacitors is widely applied because of their simple synthesis, good electronic conductivity, and unique electrochemical properties [5,6,7]. Out of all the conducting polymers studied, polypyrrole (PPy) has prominently demonstrated to hold impressive conductivity and electrochemical behavior. Nanostructured PPy composites can be generally synthesized in various ways, such as by in situ chemical/electrochemical oxidation [8,9,10], reactive template methods [11,12], interfacial polymerization [13,14,15], electrospinning [16,17], and so on. It is important to note that preparation strategy and morphology control are crucial in fabricating efficient supercapacitors [18,19,20,21,22]. Various PPy nanocomposites were prepared, for example, PPy/carbon [8,9,23], PPy/metallic element [24,25,26,27], PPy/inorganic clay [28,29], or PPy/organic nanocomposites [30,31,32]. Thus far, among these PPy derivatives, PPy/carbon nanocomposites have been extensively studied for supercapacitor devices, wherein their capacitance ranges from 100 to 600 F g^−1^ [33,34].

Recently, Minisy et al. [35] showed a new approach to preparing highly conductive PPy. It was discussed that neat PPy polymerized in frozen-state conditions (−24 °C) has better conductivity (24 S cm^−1^) compared to PPy synthesized at room temperature (5 S cm^−1^) [36]. Moreover, organic dyes (such as methyl red [37], safranin [38,39], methyl orange (MO) [40,41,42], Acid Blue 25 (AB) [43,44,45], etc.) were studied in various ways as templates or additives during the chemical or electrochemical polymerization of pyrrole and yielded improved PPy conductivity and electroactivity. AB and MO organic dyes were used as structural directing agents, which is based on different mechanisms. They can create hard templates that direct the morphology of PPy into 1D nanostructures [41,43,45,46,47]. When PPy was polymerized in frozen-state conditions (−24 °C) with organic dyes present, its conductivity was further enhanced, reaching 109 S cm^−1^ for PPy with MO and 150 S cm^−1^ for PPy with AB [36]. Along with the PPy conductivity increase, when MO was added during the polymerization of PPy, PPy nanotubes were obtained. In the presence of AB, the morphological formation of PPy nanofibers was observed (see Figure S1). The freezing polymerization method has produced nanostructured PPy with enhanced physicochemical properties. Therefore, frozen-state polymerized PPy with and without the presence of MO and AB showed outstanding properties that are very valuable for energy storage systems, such as for supercapacitors.

Herein, we continued to further investigate the electrochemical properties of frozen-state polymerized PPy with and without the addition of MO and AB. We specifically studied the electroactivity, capacitance in various aqueous electrolytic media (HCl, H_2_SO_4_, KCl, and KOH solutions), and electrochemical stability of PPy by cyclic voltammetry (CV), galvanostatic charge–discharge (GCD), and electrochemical impedance spectroscopy (EIS). Moreover, a symmetrical flexible supercapacitor prototype was fabricated to test the supercapacitive behavior of the nanostructured PPy.

## 2. Materials and Methods

### 2.1. PPy-Based Electrode Preparation

PPy without dyes (PPy-FS) and in the presence of MO (PPy-MO) and AB (PPy-AB) were prepared via frozen-state chemical oxidative polymerization. The detailed procedures for preparing all the forms of PPy under frozen conditions and at room temperature were previously described in detail in the study by Minisy et al. [36]. In this method, PPy-MO was prepared by the oxidative polymerization of 0.15 M pyrrole with 0.3 M iron(III) chloride and 75 mM MO. PPy-AB was prepared by the oxidation of 0.2 M pyrrole with 0.5 M iron(III) chloride and 10 mM AB. PPy-FS (without dye) was prepared by mixing 0.2 M pyrrole with 0.5 M iron(III) chloride solutions. The optimal conditions of each reaction were previously studied separately in order to obtain the highly conductive PPy nanostructures [36,41,43]. The dyes were thoroughly washed out of the PPy powder by excessive rinsing with 0.2 M HCl and ethanol. All the reactions were carried out at −24 °C. Additionally, PPy without dyes was polymerized at room temperature by mixing 0.2 M pyrrole with 0.5 M iron(III) chloride solutions and was used as a reference (PPy-RT). PPy-FS, PPy-MO, PPy-AB, and PPy-RT were used as the pseudocapacitive components of the electrode. 

A total of 10 mg of each as-prepared active materials (PPy-FS, PPy-MO, PPy-AB, and PPy-RT) was dispersed in 1 mL of water–isopropanol mixture consisting of 50 µL of Nafion 117 solution (contains ~5% in a mixture of lower aliphatic alcohols and water purchased from Sigma-Aldrich, Burlington, MA, USA), 400 µL of isopropanol, and 550 µL of de-ionized water. Then, 1 µL of suspension was drop cast onto a 3 mm diameter glassy carbon (GC) electrode and dried in the air for at least 15 min at room temperature. The loaded mass density was 0.14 mg cm^−2^, which was calculated based on the ratio of the mass of the deposited active material (0.01 mg) to the planar area of the electrode surface (7.07 × 10^−2^ cm^2^). Basic electrochemical properties of PPy-FS, PPy-MO, PPy-AB, and PPy-RT were elucidated in a three-electrode electrochemical cell deposited on a glassy carbon (GC) electrode used as the working electrode.

### 2.2. Electrochemical and Spectroscopic Characterization

Electrochemical measurements of PPy-FS, PPy-MO, PPy-AB, and PPy-RT on GC electrodes were conducted in a three-electrode electrochemical system with a platinum rod as the counter electrode, and AgCl coated Ag wire was used as the pseudo reference electrode. Cyclic voltammograms (CV) and galvanostatic charge–discharge curves (GCD) of the PPy-based electrodes were recorded at different scan rates (10, 20, 50, 100, and 200 mV s^−1^) and at different applied current densities, J (5.0, 6.0, 7.0, 8.0, 9.0, 10.0 A g^−1^), within a potential window from −0.2 V to 0.8 V. Electrochemical impedance spectra (EIS) were measured in a frequency range from 0.1 Hz to 100,000 Hz with sinusoidal potential amplitude set at 5 mV. The potential was preserved for 15 min for equilibrium conditions before each spectrum registration. All electrochemical measurements were conducted in 0.2 M HCl, 1 M H_2_SO_4_, 2 M KCl, and 6 M KOH aqueous solution. The cycling stability test was performed through cyclic voltammetry at 100 mV s^−1^ for 5000 cycles in 0.2 M HCl solution. All electrochemical measurements were performed using Bio-Logic SAS VSP-300 potentiostat equipment. 

The gravimetric capacitances (F g^−1^), *C_CV_* and *C_GCD_*, of each PPy-based electrode were calculated from the cyclic voltammograms and charge–discharge curves using the following equations:

(1) $$C_{CV}=\frac{\int IdV}{2\times m\times ∆V\times v}$$

(2) $$C_{GCD}=\frac{I∆t}{m∆V}$$

where ∫IdV = area of voltammogram, m = mass of the active material (1.0 × 10^−5^ g), ∆V is the potential window (−0.2 V to 0.8 V), v is the scan rate (V s^−1^), and I is the applied charge–discharge current (A).

Raman spectra of PPy-FS, PPy-MO, PPy-AB, and PPy-RT on GC electrodes were obtained through the excitation lines 785, 633, and 514 nm and were recorded with a Renishaw InVia Reflex Raman microspectrometer (Wotton-under-Edge, UK). Scattered light was analyzed using a spectrograph with holographic gratings of 1200, 1800, and 2400 lines mm^−1^, respectively. A research-grade Leica DM LM microscope was used to focus the laser beam on the active material, which was fixed by a specific accessory to hold the electrode while moving the stage. A Peltier-cooled CCD detector (576 × 384 pixels) registered the dispersed light.

### 2.3. Supercapacitor Assembly

A slurry mixture was prepared consisting of 50 mg of PPy-MO, 550 µL of deionized water, 400 µL of isopropanol, and 50 µL of Nafion 117 solution. The obtained mixture was drop cast onto two symmetrical carbon cloths cut into 4 cm by 1 cm substrates and was dried in the oven at 60 °C for 3 h. The drop cast active material covered half of the area of the carbon cloth (2 cm by 1 cm). The two prepared symmetrical electrodes were sandwiched together, including a filter paper (grade: 388) soaked in 1 M H_2_SO_4_ aqueous electrolyte as a separator. The total loaded mass density of the supercapacitor was 6.6 mg cm^−2^. The assembly was then wrapped by a plastic insulator and pressed tightly, making sure that the electrolyte was trapped in the system. 

### 2.4. Supercapacitive Characterization

Electrochemical studies of the assembled supercapacitor were performed through EC–Lab software V11.20 using Bio-Logic SAS VSP-300 potentiostat equipment. Electrochemical impedance spectroscopy was carried out within a frequency ranging from 0.1 Hz to 100,000 Hz. Cyclic voltammetry studies were performed at the scan rates 10, 20, 50, 100, and 200 mV s^−1^ in a potential window from −0.2 V to 0.8 V. Galvanostatic charge–discharge measurements were obtained at an applied constant current density varying from 1.5 to 4.5 A g^−1^ in the same potential range. The gravimetric capacitance in F g^−1^ of the supercapacitor was calculated using the area of the CV curve (*C_SCV_*) and the slope of the discharge curve of the GCD (*C_SCD_*) [48]:

(3) $$C_{SCV}=k\frac{\int IdV}{m\times v\times ∆V}$$

(4) $$C_{SCD}=k\frac{I\times ∆t}{∆V\times m}$$

where ∫IdV is the integrated area of the CV curve, m is the mass of the active material in grams, v is the scan rate, I is the applied current, ∆t is the discharge time during the discharge process, ∆V is the working potential window, and k is the constant multiplier. In this study, the value of k was 4 since the masses of the active material on both electrodes were considered. The energy densities $\left(E_{S},\;Wh{kg}^{-1}\right)$ and power densities ${(P}_{S,\;\;}\;W{kg}^{-1})$ were obtained from their GCD curves using the following equations:

(5) $$E_{S}=\frac{1}{2}C_{S}({∆V)}^{2}\times \frac{1000}{3600}$$

(6) $$P_{S}=\frac{E_{S}}{∆t}=\frac{I∆V}{2m}\times 1000$$

Moreover, a cycling stability test was carried out at 10,000 charge–discharge cycles at a 3 A g^−1^ current density. The total cell resistance was determined in two ways: (1) IR_drop_ based on the potential drop on the discharge curves and (2) equivalent resistance (ESR) values based on the Nyquist plot of the device before and after 10,000 cycles.

## 3. Results and Discussion

### 3.1. PPy-Based Electrodes in Different Aqueous Electrolytes

Cyclic voltammograms of PPy-FS, PPy-MO, PPy-AB, and PPy-RT on GC electrodes were studied in different aqueous solutions (0.2 M HCl, 1 M H_2_SO_4_, 2 M KCl, and 6 M KOH) in a three-electrode system. In Figures S2–S5, the CV curves were obtained at scan rates: 10, 20, 50, 100, and 200 mV s^−1^ from −0.2 V to 0.8 V potential range. As seen in the CVs, the current density peak for each PPy-based electrode increased at an increasing scan rate. This behavior was observed in all aqueous electrolytes displaying the electroactive property of the electrodes. In particular, the obtained CVs in 0.2 M HCl, 1 M H_2_SO_4_, and 2 M KCl showed a consistent quasi-rectangular curve with symmetry in shape along the anodic and cathodic sweeps in all scan rates. These are indications that the overall capacitive behavior of the electrodes is ascribed to the contribution of both electric double-layer capacitance (EDLC) and pseudocapacitance (faradaic contribution). The current density peaks were relatively greater in acidic than in alkaline conditions, suggesting that the electroactivity of the PPy-based electrodes increased in the following order of electrolytes: HCl > H_2_SO_4_ > KCl > KOH (as shown in Figures S2–S5). The electrochemical response in acidic electrolytes rapidly increased the current density for every scan rate increase, indicating a good capacitive performance. In the KOH solution, the voltammograms were not uniform in shape at an increasing scan rate. This can be attributed to the deprotonation of PPy in the alkaline solution, which resulted in lower conductivity and instability [46,47]. Moreover, it was observed, in the KOH solution, that the electroactivity of PPy-based electrodes was at poor rate capacity due to its low current peaks. Thus, the capacitive performance in the acidic medium was better than in neutral and basic electrolytes.

**Figure 1:**
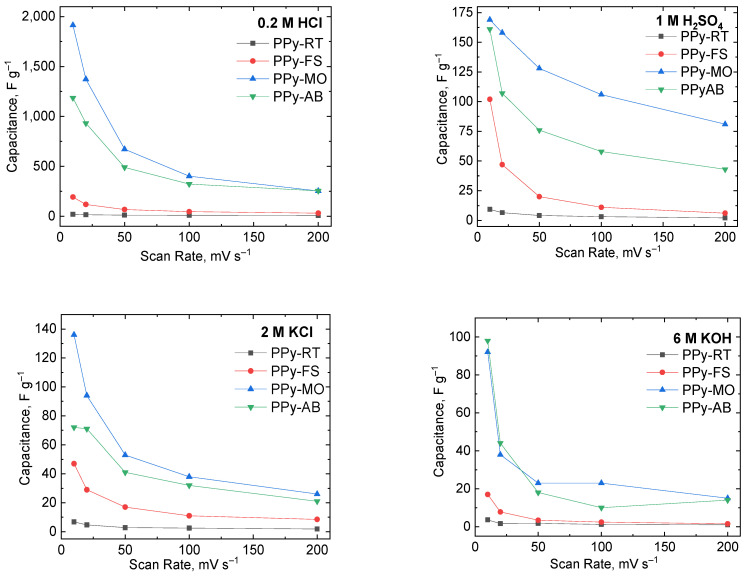
Gravimetric capacitance of PPy-FS, PPy-MO, PPy-AB, and PPy-RT measured through cyclic voltammetry in different electrolytes (0.2 M HCl, 1 M H2SO4, 2 M KCl, and 6 M KOH).

The gravimetric capacitance of PPy-based electrodes was dependent on the area under the CV curve. Therefore, the larger the area of the voltammogram, the higher the calculated capacitance. Herein, the gravimetric capacitance of the PPy-based electrodes was calculated using Equation (1) and is shown in Figure 1 and Table S1. The capacitance decreased with the increasing scan rate since the diffusion time of charges/ions to the active material is faster at higher scan rates. The highest calculated capacitance was estimated at 10 mV s^−1^ in all electrolytic solutions. The determined capacitance in the HCl solution was the highest, followed by the capacitance values obtained in the H_2_SO_4_, KCl, and KOH solutions. Although both HCl and H_2_SO_4_ are acidic electrolytes, their current response differs as their ionic size differs. Cl^−^ ions are faster and easier to move across the diffusion path compared to SO_4_^2−^ ions; hence, capacitance was higher in the HCl solution.

**Figure 2:**
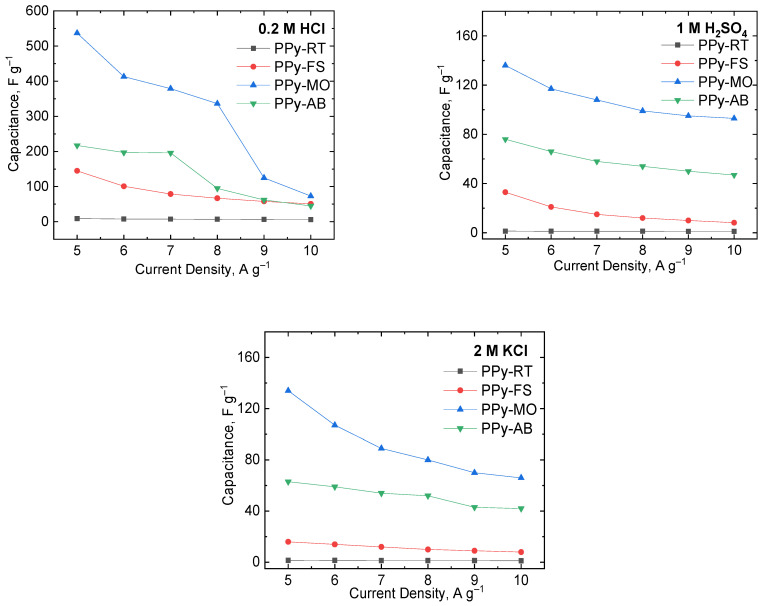
Gravimetric capacitance of PPy-FS, PPy-MO, PPy-AB, and PPy-RT measured through galvanostatic charge–discharge in 0.2 M HCl, 1 M H_2_SO_4_, and 2 M KCl electrolytic solution.

The GCD curves in Figures S6–S9 of PPy-based electrodes in all electrolytes exhibited a non-symmetric charge–discharge process for every applied current density. This was ascribed to the pseudocapacitive nature of PPy, which was cohesive with the CV results. The gravimetric capacitance of PPy-based electrodes was also calculated based on the discharge process at varying current densities using Equation (2). The capacitance values estimated from GCD curves are presented in Figure 2 and Table S2. The gravimetric capacitance values from the discharge curves exhibited a similar trend, wherein higher capacitance values were computed in acidic electrolytes. The CV and GCD curves proved the excellent electroactivity of PPy-based electrodes in acidic electrolytes. Hence, the electrochemical behavior of PPy-based electrodes in the acidic medium was better than in basic solutions owing to the high conductivity and fast charge transfer behavior of PPy.

### 3.2. The Frozen-State Polymerized PPy with Dyes and Its Capacitive Performance

Dye-incorporated PPy prepared in the frozen state displayed the formation of homogenous PPy nanostructures (check Figure S1), which greatly influenced the improvement of PPy’s electrical conductivity. In this study, an in-depth investigation of the electrochemical properties of frozen-state polymerized PPy in the presence of organic dyes (MO and AB) was carried out. The calculated capacitance values of PPy-FS, PPy-RT, PPy-MO, and PPy-AB are presented in Tables S1 and S2 of the Supporting Information File.

In HCl solution, PPy-MO was observed to have the highest capacitance (1914 F g^−1^), followed by PPy-AB (1186 F g^−1^), PPy-FS (193 F g^−1^), and PPy-RT (20 F g^−1^) at 10 mV s^−1^. Also, a similar course of electrochemical response was noticed based on PPy’s computed capacitance from GCD curves at 5 A g^−1^: PPy-MO (537 F g^−1^); PPy-AB (217 F g^−1^); PPy-FS (145 F g^−1^); and PPy-RT (9.1 F g^−1^). This large improvement in the capacitance of PPy was due to its morphological change caused by MO and AB. The presence of MO and AB modified the PPy morphology from globules to uniform nanostructures, as presented in Figure 1. This alteration of PPy’s structural arrangement had a significant effect on PPy’s capacitance. The interconnected structure and more uniform porosity in PPy-MO and PPy-AB promoted higher chances for the charges/ions to access the electrode surface at a shorter diffusion time. This was clearly evident on the higher current density peaks achieved by PPy-MO and PPy-AB compared to PPy-FS and PPy-RT. Moreover, PPy-MO and PPy-AB contain more electrochemically active sites, enabling full access for charges/ions to move from the electrolyte to the electrode, thereby resulting in a longer discharging process and higher gravimetric capacitance. By comparing the gaps in the capacitance values of PPy-MO and PPy-AB, this can be attributed to their differences in morphology, as PPy-MO contains nanotubular structures while PPy-AB has a nanofibrillar structural arrangement. 

The capacitance of CV curves of PPy-MO at 10 mV s^−1^ (169 F g^−1^) and PPy-AB (161 F g^−1^) in H_2_SO_4_ solution had a similar trend when compared to pristine PPy prepared in the frozen state (102 F g^−1^) and at room temperature (9.3 F g^−1^). Likewise, the calculated PPy-MO capacitance (136 F g^−1^) at 5 A g^−1^ from GCD curves showed to be the highest. PPy-AB is next, having a capacitance of 76 F g^−1^, followed by PPy-FS (33 F g^−1^) and PPy-RT (1.2 F g^−1^) measured at 5 A g^−1^. This large increase in capacitance indicates the improved electrochemical behavior of PPy in the H_2_SO_4_ electrolyte. It was noted from previous works that using H_2_SO_4_ as an electrolyte for supercapacitors has led to higher capacitance with smaller equivalent series resistance (ESR) values because of its good ionic conductivity [49,50,51]. Hence, this study opted to test the electrochemical properties of PPy with and without the presence of dyes in H_2_SO_4_ solution for supercapacitor evaluation.

PPy-MO and PPy-AB were also experimented with a 2 M KCl solution. A large gap in the calculated capacitance from CV curves at 10 mV s^−1^ of PPy-MO (136 F g^−1^) and PPy-AB (72 F g^−1^) to the capacitance of PPy-FS (47 F g^−1^) and PPy-RT (6.8 F g^−1^) was observed. Similar behavior from charge–discharge curves at 5 A g^−1^ were seen for the computed capacitance of PPy-MO (134 F g^−1^), PPy-AB (63 F g^−1^), PPy-FS (16 F g^−1^), and PPy-RT (1.2 F g^−1^). As expected, even in a neutral solution, the capacitive behavior of PPy-MO and PPy-AB was excellent. This certainly proves that modifying the processability of preparing PPy has a positive outcome in enhancing its electrochemical properties. 

Furthermore, the electrochemical performance of PPy-MO and PPy-AB in KOH solution was tested. In this electrolyte, PPy-MO and PPy-AB have relatively high peak currents compared to PPy-FS and PPy-RT based on their CV curves. The capacitance values of PPy-MO vary between 15 F g^−1^ and 92 F g^−1^ and between 14 F g^−1^ and 98 F g^−1^ for PPy-AB measured from 10 mV s^−1^ to 200 mV s^−1^. This implies that, in alkaline conditions, PPy-MO and PPy-AB have good electroactivity. In spite of their good current response, the CVs are not stable or symmetric with the increase in scan rate. This can be attributed to the loss of conductivity of PPy in basic electrolytes. For this reason, charge–discharge measurements in KOH electrolytes were omitted. 

**Figure 3:**
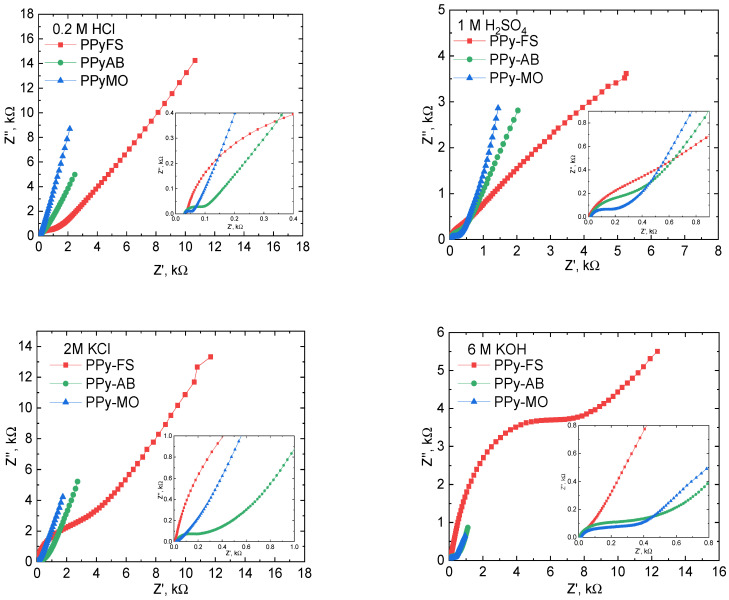
Impedance spectra of PPy and PPy with dyes prepared in the frozen state in 0.2 M HCl, 2 M KCl, 6 M KOH, and 1 M H_2_SO_4_.

EIS studies of PPy-FS, PPy-MO, and PPy-AB in various electrolytes were evaluated as well. In Figure 3, the Nyquist plots of PPy-based electrodes showed different spectral representations in different electrolytes. Semicircles were present in the high frequency region, indicating the charge transfer process taking place between the working electrode and the electrolyte [52]. Straight lines of varying slopes were seen in the low frequency region, referring to the diffusion of charges/ions in the electrodes [52,53]. Here, PPy-MO had the least charge transfer resistance, R_ct_ (see Table S3), in all electrolytes, indicating its good conductivity, and then, it was succeeded by PPy-AB and PPy-FS. In the lower frequency region, the increase in the slope of the line refers to the increase of capacitive behavior of PPy. The Nyquist plots showed that the capacitance increased in the following order: PPy-MO > PPy-AB > PPy-FS, according to the increase of their slopes. Thus, we verified that the frozen-state polymerized PPy with the presence of dyes enhanced the overall electrochemical properties of PPy. Moreover, the collected data from Nyquist plots were fitted to obtain the equivalent electrochemical circuit (EEC) presented in Figure S10 and Table S4. The fit corresponds to the electrochemical processes occurring on the PPy-based electrode at this potential. Based on the acquired EEC, R_1_ represents the total resistance of the electrode, which includes the bulk electrolyte and electrode resistances [54]. R_2_ represents R_ct_, which corresponds to the charge transfer resistance at a lower frequency. R_2_ indicates the resistance involved during the diffusion of charges/ions between the polymer and electrolyte interface that usually occurs in the lower frequency region [54,55]. The presence of constant phase elements, CPE_1_ and CPE_2,_ in EEC reflects the non-ideal capacitive behavior of the PPy-based electrode [56]. Its underlying physical basis can be attributed to the double-layer capacitive behavior of charges on an inhomogeneous electrode surface [56,57,58]. The inhomogeneity on the electrode surface may be caused by the microscopic surface roughness, porosity, nature of the electrode, and the dynamic disorder associated with diffusion [57,58]. The W in EEC refers to Warburg element, indicating the diffusion impedance of charges caused by the faradaic processes involved on the PPy-based electrode [54,58]. 

**Figure 4:**
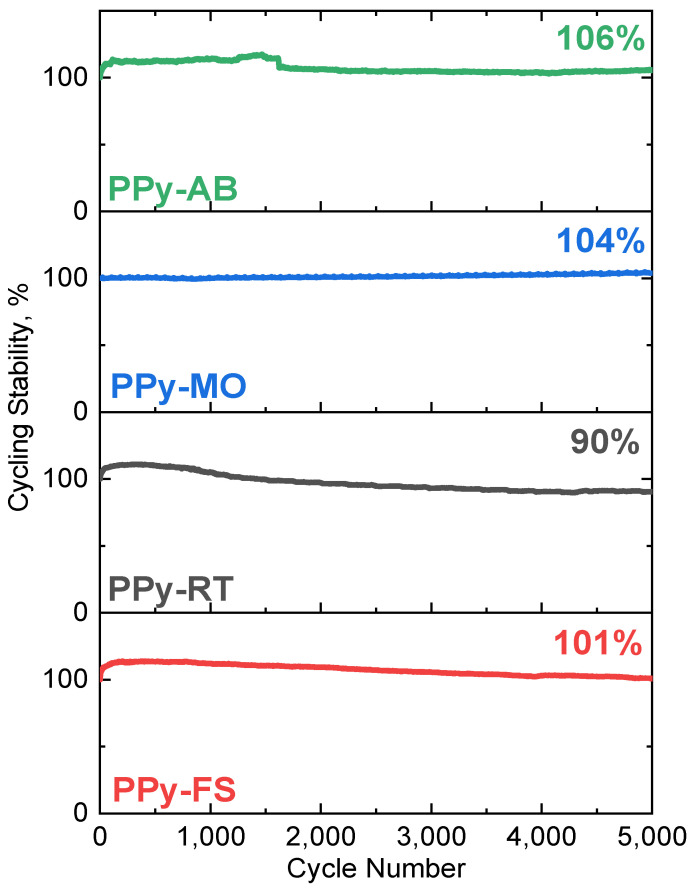
Cycling stability of PPy-FS, PPy-RT, PPy-MO, and PPy-AB for 5000 cycles tested in 0.2 M HCl solution.

The capacitance retention of electrodes was measured through cyclic voltammetry in a three-electrode system (see Figure 4). In 5000 potential cycling, steady gain of capacitance was observed for all frozen-state polymerized PPy with and without the dyes added. PPy-FS (101%), PPy-MO (104%), and PPy-AB (106%) displayed outstanding stability that is very valuable for supercapacitor use. 

### 3.3. Spectroscopy Study

**Figure 5:**
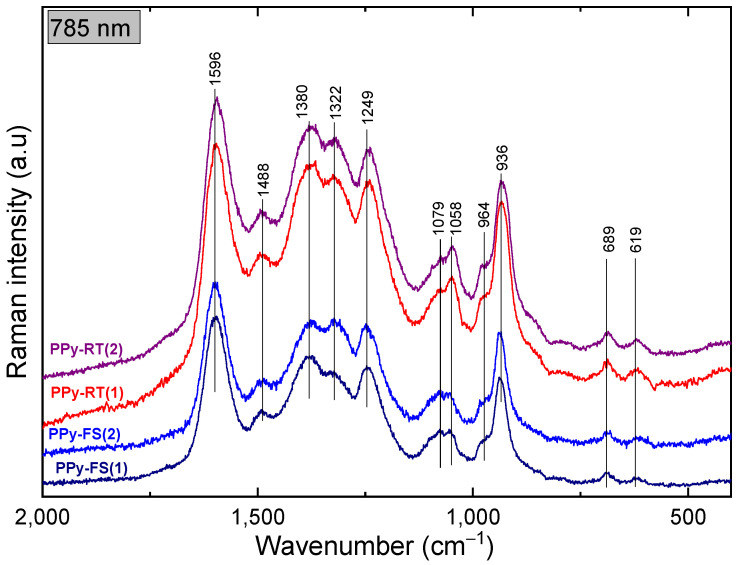
Raman spectra of PPy-RT and PPy-FS before (1) and after (2) the cycling stability test at 785 nm excitation.

The molecular structures of PPy-RT, PPy-FS, PPy-MO, and PPy-AB before and after cycling stability tests were assessed by Raman spectroscopy. In Figure 5, the spectral features of PPy can be clearly distinguished and are in accordance with the previous reports [46,59]. The Raman spectrum of PPy-FS exhibited an increase in the intensity of the band at 1079 cm^−1^ (C–H deformation in bipolaron) and a decrease in the intensity of the band at 1058 cm^−1^ (C–H deformation in polaron) as compared to the spectrum of PPy-RT. This corresponds to the increased protonated state of PPy-FS [60]. After cycling stability tests, a significant change in the intensities of the two peaks 1380 cm^−1^ and 1322 cm^−1^, which are attributed to the inter-ring stretching C–C vibration mode of PPy in bipolaron and polaron structures, respectively, was noticed in the spectrum of PPy-FS. This is probably because of the irreversible oxidation state of PPy-FS during electrochemical cycling.

**Figure 6:**
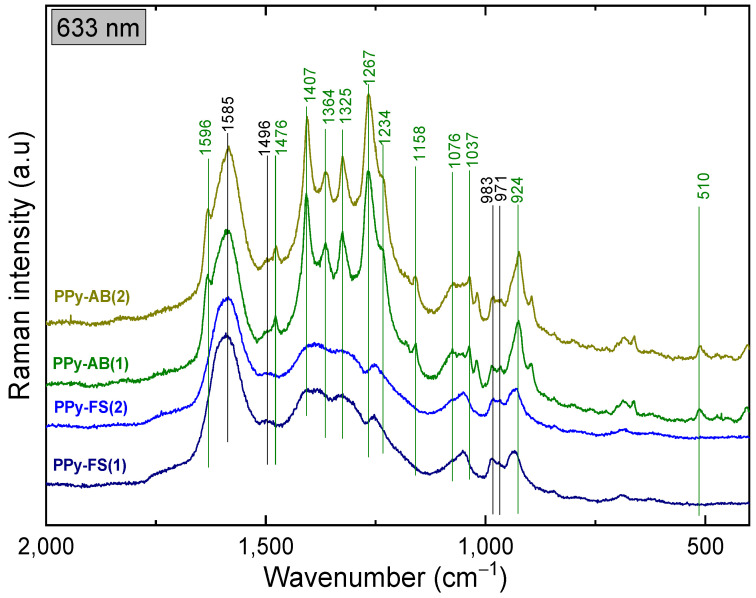

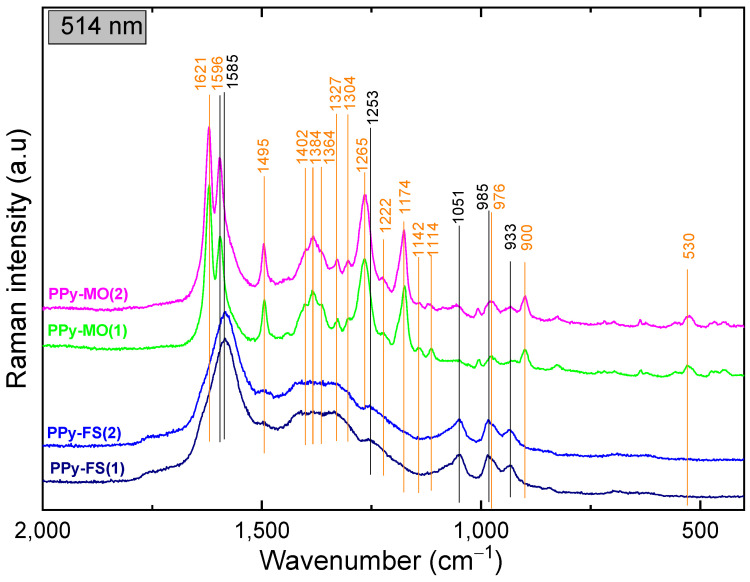
Raman spectra of PPy-FS, PPy-AB, and PPy-MO before (1) and after (2) cycling tests at the 633 nm and 514 nm excitation lines, respectively.

The Raman spectra of PPy-AB before and after the cycling stability test are given in Figure 6. The green solid lines refer to AB peaks and the black ones to PPy-FS characteristic peaks. Typical bands of AB dye molecule at 1407 cm^−1^, 1364 cm^−1^, 1325 cm^−1^, and 1267 cm^−1^ are related to C=C and C–N stretching vibrations. Other peaks refer to the deformation vibrations of AB molecules located at 1158 cm^−1^, 1037 cm^−1^, 924 cm^−1^, and 510 cm^−1^ [45]. In addition, the small shoulder appearing at 1596 cm^−1^ in the PPy-AB spectrum belongs to C=C stretching in the bipolaron structure, which indicates a higher protonation state of PPy in the presence of the dye.

For PPy-MO, the orange solid lines refer to MO distinctive spectral peaks, and the black ones refer to the spectral features of PPy-FS. Raman spectra of PPy-MO were measured using a 514 nm excitation line since it is in resonance with the energetic transitions in MO. The following are the detected vibrational features of MO: 1621 cm^−1^ (N–H in-plane deformation), 1495 cm^−1^ (N–ring stretching), 1265 cm^−1^ (C–N stretching, N–H stretching), 1174 cm^−1^ (N–N bending), 1114 cm^−1^ (S–O stretching), 1142 cm^−1^ (C–S stretching), 1364 cm^−1^ (S–ring and C–C stretching), 1384 cm^−1^ (C–SO_2_–O) stretching), and 1402 cm^−1^ (N=N stretching) [42].

After long potential cycling, there were no Raman shifts in the spectral bands of PPy-MO and PPy-AB. Therefore, the stability of the molecular structure of PPy was improved in the presence of MO and AB. 

### 3.4. Supercapacitor Assembly

**Figure 7:**
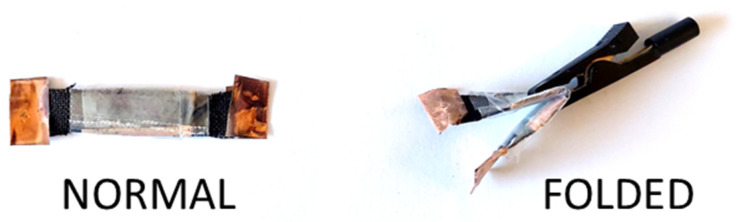
Assembled flexible supercapacitor in normal and folded orientations.

A flexible supercapacitor device was constructed using the as-prepared PPy-MO as the active material for the two-electrode cell. PPy-MO was chosen since it has the best electrochemical performance among the rest of the PPy-based materials studied here. The assembled flexible supercapacitor was examined in two positions, (1) in normal and (2) folded orientations (see Figure 7), in order to test the supercapacitive behavior of the device when its orientation varied. Different electrochemical methods (CV, GCD, EIS, and stability measurements) were conducted to study the prototype. 

**Figure 8:**
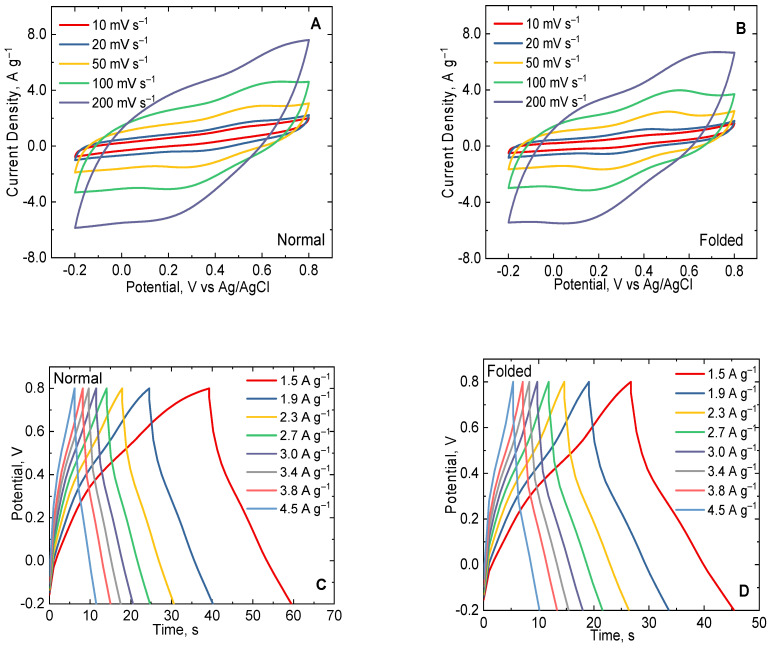
(**A**,**B**) CV curves of the PPy-MO-based supercapacitor device (in normal and folded positions) measured at 10, 20, 50, 100, and 200 mV s^−1^ scan rates. (**C**,**D**) GCD curves of the normal and folded PPy-MO-based supercapacitor device at varying current densities from 1.5 A g^−1^ to 4.5 A g^−1^.

Figure 8A,B shows the CV curves of the supercapacitor device at different scan rates (10, 20 50, 100, and 200 mV s^−1^) in normal and folded positions. The voltammograms showed a high current response to the quasi-rectangular shape similar to the CVs of PPy-MO obtained in a three-electrode system. The increase of the current with respect to scan rate evidently confirmed the outstanding electrochemical activity of the PPy-MO-based supercapacitor. The CV curve in the folded position was on a par with the normal one, indicating the good stability of the constructed device. At scan rates above 50 mV s^−1^, the charge transfer process became faster, which limited the diffusion of charges/ions following the deviation of CVs from the quasi-rectangular shape. Galvanostatic charge–discharge measurements were performed at an applied current density that varied from 1.5 A g^−1^ to 4.5 A g^−1^, as shown in Figure 8C,D. The GCD curves of the device in both orientations illustrate a non-symmetric triangular form akin to the GCD curves observed in the three-electrode system.

The capacitance of the device was determined using Equations (3) and (4), respectively. As shown in Table 1, the decrease in capacitance with respect to the increase in scan rate was caused by the high current response demonstrated by the PPy-MO-based supercapacitor. The highest computed capacitance was at 10 mV s^−1^, equivalent to 122 F g^−1^ in the normal position, and it slightly decreased to 105 F g^−1^ when folded. Likewise, as the applied current density increased, the capacitance decreased as well because of the fast charge–discharge performance of the device. At the applied current density of 1.5 A g^−1^, the capacitance was 116 F g^−1^ (in normal position), and the capacitance lowered to 107 A g^−1^ in the folded state. The capacitance difference in both orientations was certainly observed since the charge transfer process changed as the device shifted positions. Yet, there was no significant disparity between the capacitive behavior of the folded and unfolded device, as the capacitance values remained in the same order of magnitude. This proves that the PPy-MO-based supercapacitor has good charge storage characteristics with good flexibility. 

**Figure 9:**
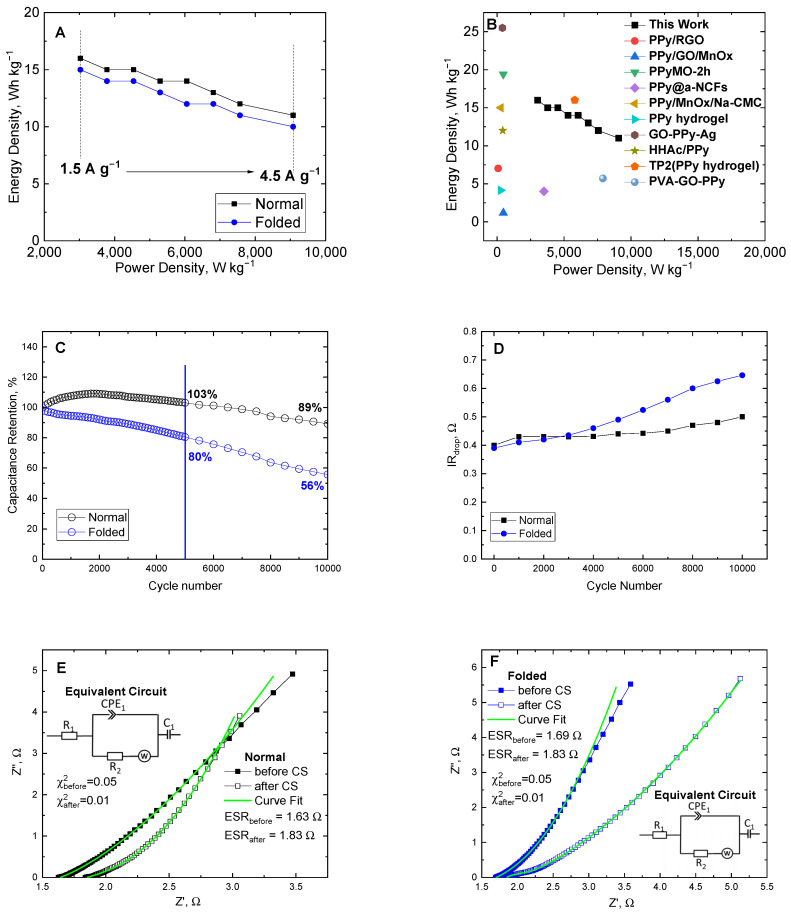
(**A**) Ragone plot of the PPy-MO-based supercapacitor in normal and folded orientation. (**B**) Comparison of the Ragone plots of the PPy-MO-based supercapacitor with other PPy-based symmetric supercapacitors (PPy/RGO [61], PPy/GO/MnOx [62], PPyMO-2h [63], PPy@a-NCFs [64], PPy/MnOx/Na-CMC [65], PPy hydrogel [66], GO-PPy-Ag [67], HHAc/PPy [68], TP2(PPy hydrogel) [69], PVA-GO-PPy [70]). (**C**) Cycling stability and (**D**) IR_drop_ behavior of the normal and folded PPy-MO-based supercapacitor devices. (**E**,**F**) EIS spectra before and after cycling stability (CS) of the normal and folded PPy-MO-based supercapacitor devices.

The energy density, power density, and Ragone plot of the PPy-MO-based supercapacitor are provided in Figure 9A and Table 1. The energy density and power density of the device in normal form were 16 Wh kg^−1^ and 3026 W kg^−1^ at 1.5 A g^−1^, respectively. The energy density lowered to 15 Wh kg^−1^ when the device was folded at 1.5 A g^−1^. Also, the power density reached up to 9077 W kg^−1^ at 4.5 A g^−1^. The measured energy and power densities of the PPy-MO-based supercapacitor were higher than or comparable to the recently reported PPy-based symmetric supercapacitors [60,61,62,63,64,65,66,67,68,69,70], as illustrated in Figure 9B. The capacitance retention of the device was estimated for 10,000 charge–discharge cycles. Figure 9C shows the capacitance retention of the PPy-MO-based device in normal and folded position. After 10,000 cycles, the capacitance of the normally positioned device was retained at 89%, and only a small increase in IR_drop_ was noticed as shown in Figure 9D. When folded, the capacitance retention was down to 56%, with a larger increase in IR drop after long cycling. In addition, the capacitance declined faster beyond 5000 cycles for both positions as a result of the drying of the electrolyte during the entire measurement period. The comparison of the capacitance retention and ESR values of the PPy-MO-based supercapacitor to other PPy-based supercapacitors was presented in Table S5. The assembled PPy-MO-based device showed good and comparable stability and ESR values, contrary to other PPy-based materials. 

Moreover, ESR values of the device were also determined before and after its cycling stability experiment (see Figure 9E,F). The ESR value of the device in the normal position was initially low (1.63 Ω), and after 10,000 cycles, its ESR slightly increased to 1.84 Ω. When folded, its ESR was at 1.69 Ω then increased marginally to 1.83 Ω after long cycling. These observations were consistent with the IR_drop_ increase after cycling stability. In addition, the small ESR difference between before and after cycling stability revealed the good electrical and ionic conductivity of the supercapacitor, regardless of the magnitude of its capacitance. Nonetheless, the stability and energy storage properties of PPy-MO can be considered competent for improvising an efficient flexible supercapacitor. 

## 4. Conclusions

This work demonstrated the electrochemical properties of PPy prepared with and without the presence of MO and AB in frozen conditions (−24 °C) and its applicability for supercapacitors. PPy-MO presented remarkable capacitive features, including the highest gravimetric capacitance of 1914 F g^−1^ at 10 mV s^−1^ and 537 F g^−1^ at 5.0 A g^−1^ in the HCl aqueous electrolyte. The Raman spectra of frozen-state polymerized PPy-FS, PPy-MO, and PPy-AB manifested a stable molecular arrangement even after 5000 potential cycling. The excellent electrochemical performances are ascribed to the nanostructured morphological network of PPy-MO and PPy-AB, giving rise to the increased electrolyte-accessible active areas. For this reason, faster charge transfer and ion diffusion were in effect, promoting the improved energy storage capabilities of PPy. In addition, a constructed PPy-MO-based supercapacitor device displayed a maximum of 122 F g^−1^ capacitance at 10 mV s^−1^ and 116 F g^−1^ at 1.5 A g^−1^. The device provided a good capacitance retention rate of 89% with low ESR values, indicating good rate capability and conductivity. These positive results serve as an indication that modifying the processability and morphology of PPy can be the groundwork to fulfill the demands of an efficient energy storage system.

## Figures and Tables

**Table 1:** Estimated capacitance, energy density, and power density of the PPy-MO-based supercapacitor device at varying scan rates and current densities.

v, mV s^−1^	C, F g^−1^		J, A g^−1^	C, F g^−1^		E, Wh kg^−1^		P, W kg^−1^
	Normal	Folded		Normal	Folded	Normal	Folded	
10	122	105	1.5	116	107	16	15	3026
20	120	106	1.9	111	102	15	14	3782
50	106	97	2.3	106	97	15	14	4539
100	89	83	2.6	102	93	14	13	5295
200	64	62	3.0	97	88	14	12	6052
			3.4	93	83	13	12	6808
			3.8	88	79	12	11	7565
			4.5	78	69	11	10	9077

## Data Availability

Data will be available upon request.

## Supplementary Materials

The following supporting information can be downloaded at: https://www.mdpi.com/article/10.3390/polym15204140/s1, Figure S1: SEM micrographs of polypyrrole prepared by frozen-state polymerization: PPy globules without dyes (PPy-FS); PPy nanotubes in the presence of MO (PPy-MO); and PPy nanofibers in the presence of AB (PPy-AB). Figure S2: CV curves of PPy-FS, PPy-RT, PPy-AB and PPy-MO measured in 0.2 M HCl solution at varying scan rates: 10, 20, 50, 100 and 200 mV s^−1^. Figure S3: CV curves of PPy-FS, PPy-RT, PPy-AB and PPy-MO measured in 1 M H_2_SO_4_ solution at varying scan rates: 10, 20, 50, 100 and 200 mV s^−1^. Figure S4: CV curves of PPy-FS, PPy-RT, PPy-AB and PPy-MO measured in 2 M KCl solution at varying scan rates: 10, 20, 50, 100 and 200 mV s^−1^. Figure S5: CV curves of PPy-FS, PPy-RT, PPy-AB and PPy-MO measured in 6 M KOH solution at varying scan rates: 10, 20, 50, 100 and 200 mV s^−1^. Figure S6: GCD curves of PPy-FS measured in 0.2 M HCl, 1 M H_2_SO_4_ and 2 M KCl solution at varying applied current density, J: 5.0, 6.0, 7.0, 8.0, 9.0 and 10.0 A g^−1^. Figure S7: GCD curves of PPy-RT measured in 0.2 M HCl, 1 M H_2_SO_4_ and 2 M KCl solution at varying applied current density, J: 5.0, 6.0, 7.0, 8.0, 9.0 and 10.0 A g^−1^. Figure S8: GCD curves of PPy-AB measured in 0.2 M HCl, 1 M H_2_SO_4_ and 2 M KCl solution at varying applied current density, J: 5.0, 6.0, 7.0, 8.0, 9.0 and 10.0 A g^−1^. Figure S9: GCD curves of PPy-MO measured in 0.2 M HCl, 1 M H_2_SO_4_ and 2 M KCl solution at varying applied current density, J: 5.0, 6.0, 7.0, 8.0, 9.0 and 10.0 A g^−1^. Figure S10: Impedance spectra of PPy and PPy with dyes prepared in frozen-state in 0.2 M HCl, 2M KCl, 6 M KOH, and 1 M H2SO4 and its corresponding equivalent circuit. Table S1: Computed capacitances of PPy-RT, PPy-FS, PPy-MO and PPy-AB in different aqueous solutions (0.2 M HCl, 1 M H_2_SO_4_, 2 M KCl and 6 M KOH) based from the CV curves obtained at different scan rates (10, 20, 50, 100 and 200 mV s^−1^). Table S2: Computed capacitances of PPy-RT, PPy-FS, PPy-MO and PPy-AB in different aqueous solutions (0.2 M HCl, 1 M H_2_SO_4_ and 2 M KCl) based from the GCD curves obtained at different applied current density, J (5.0, 6.0, 7.0, 8.0, 9.0 and 10.0 A g^−1^). Table S3: R_s_ and R_ct_ values based from the physical interpretation of the EIS spectra of PPy-FS, PPy-MO and PPy-AB in 0.2 M HCl, 1 M H_2_SO_4_, 2 M KCl and 6 M KOH aqueous electrolytes measured in three-electrode system. Table S4: Estimated fitting parameters based on the fitted equivalent circuit. Table S5. Comparison in capacitance retention and ESR values of the assembled PPy-MO-based supercapacitor to other PPy-based supercapacitors.
