# Supplementary material for: Highly Electroactive Frozen-State Polymerized Polypyrrole Nanostructures for Flexible Supercapacitors
Source: Polymers (Basel). 2023 Oct 18;15(20):4140. doi: 10.3390/polym15204140 (PMC10610487; doi:10.3390/polym15204140)
Supplement: Supplementary file 1 [file polymers-15-04140-s001.zip › polymers-2660009-supplementary.pdf]

SUPPORTING INFORMATION

1. Scanning Electron Microscopy (SEM)

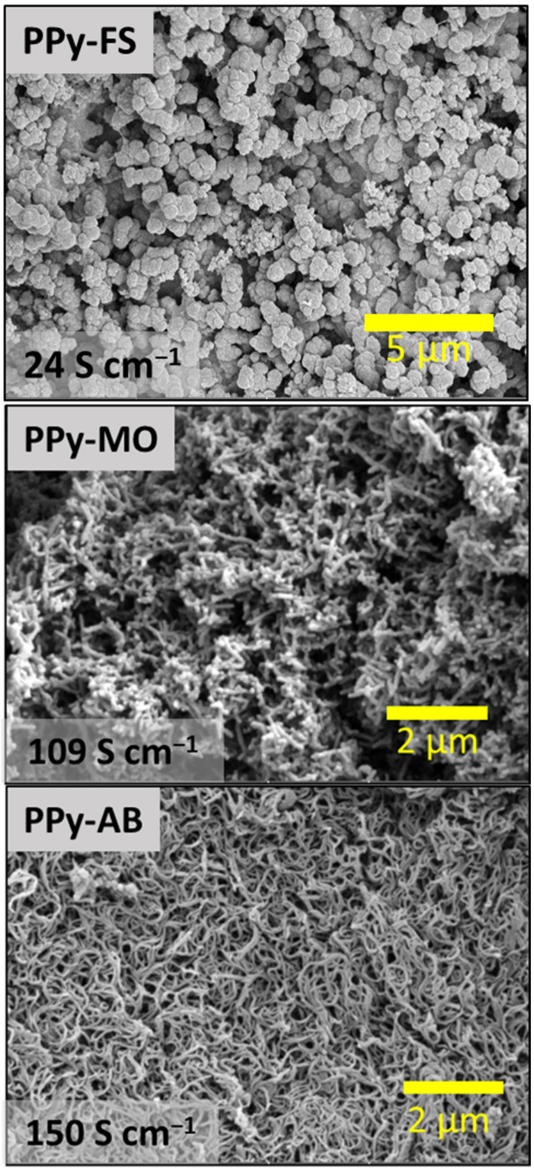

Figure S1. SEM micrographs of polypyrrole prepared by frozen-state polymerization: PPy globules without dyes (PPy-FS); PPy nanotubes in the presence of MO (PPy-MO); and PPy nanofibers in the presence of AB (PPy-AB) [Minisy, I.M.; Acharya, U.; Kobera, L.; Trchová, M.; Unterweger, C.; Breitenbach, S.; Brus, J.; Pfleger, J.; Stejskal, J.; Bober, P. Highly conducting 1-D polypyrrole prepared in the presence of safranin. J. Mater. Chem. C 2020 8 12140-12147. https://doi.org/10.1039/D0TC02838J; Minisy, I.M.; Bober, P. Frozen-State Polymerization as a Tool in Conductivity Enhancement of Polypyrrole. Macromol. Rapid Commun. 2020 41 2000364. https://doi.org/10.1002/marc.202000364].

2. Cyclic Voltammograms

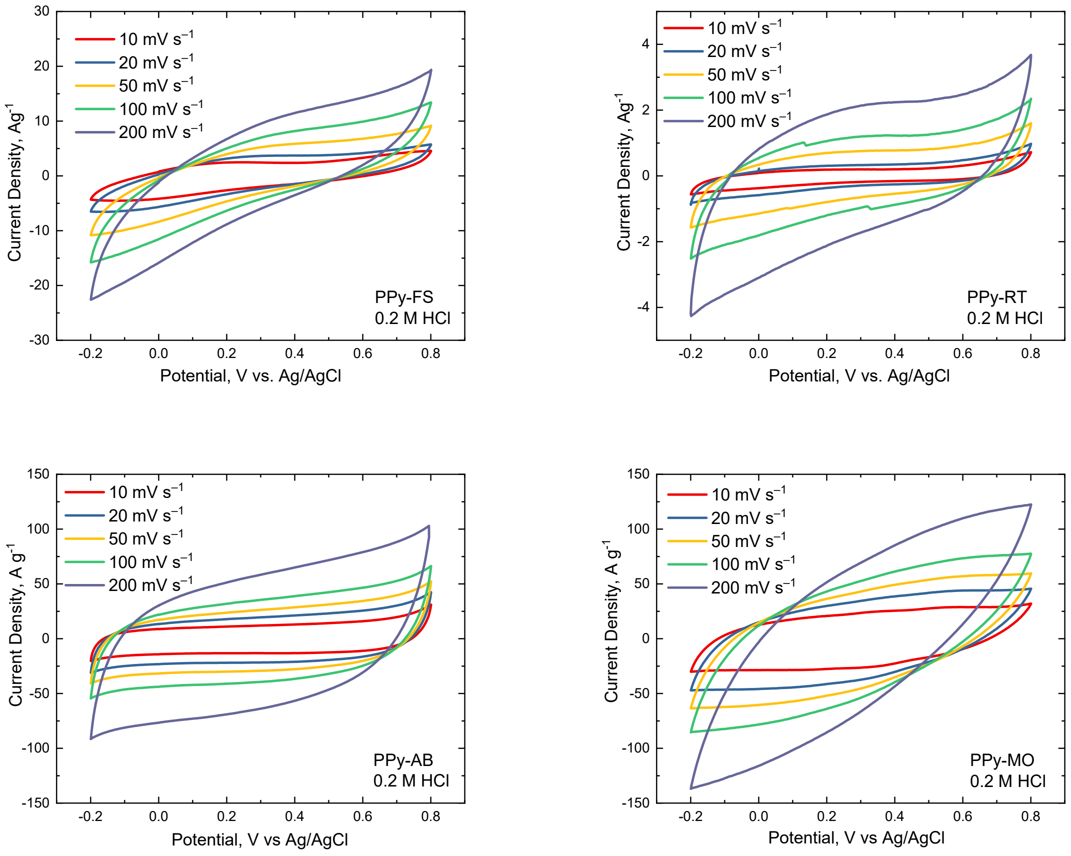

Figure S2. CV curves of PPy-FS, PPy-RT, PPy-AB and PPy-MO measured in 0.2 M HCl solution at varying scan rates: 10, 20, 50, 100 and 200 mV s^{-1}.

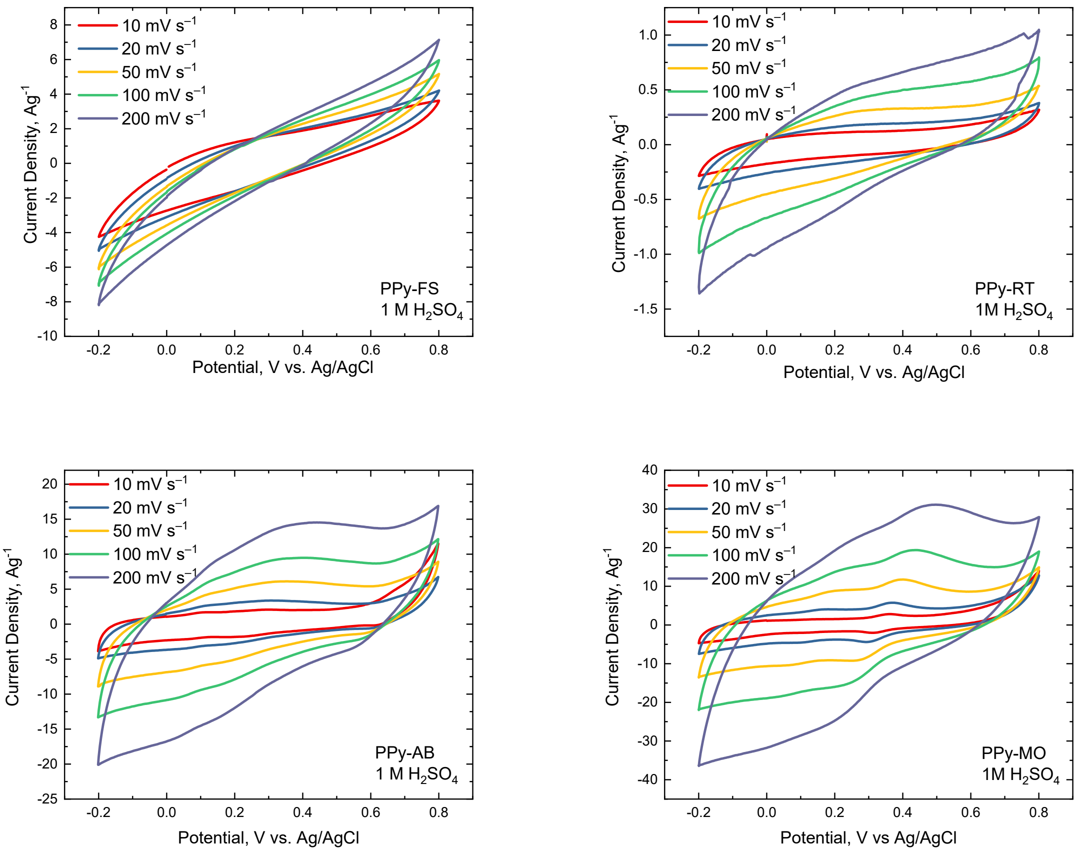

Figure S3. CV curves of PPy-FS, PPy-RT, PPy-AB and PPy-MO measured in 1 M H_2SO_4 solution at varying scan rates: 10, 20, 50, 100 and 200 mV s^{-1}.

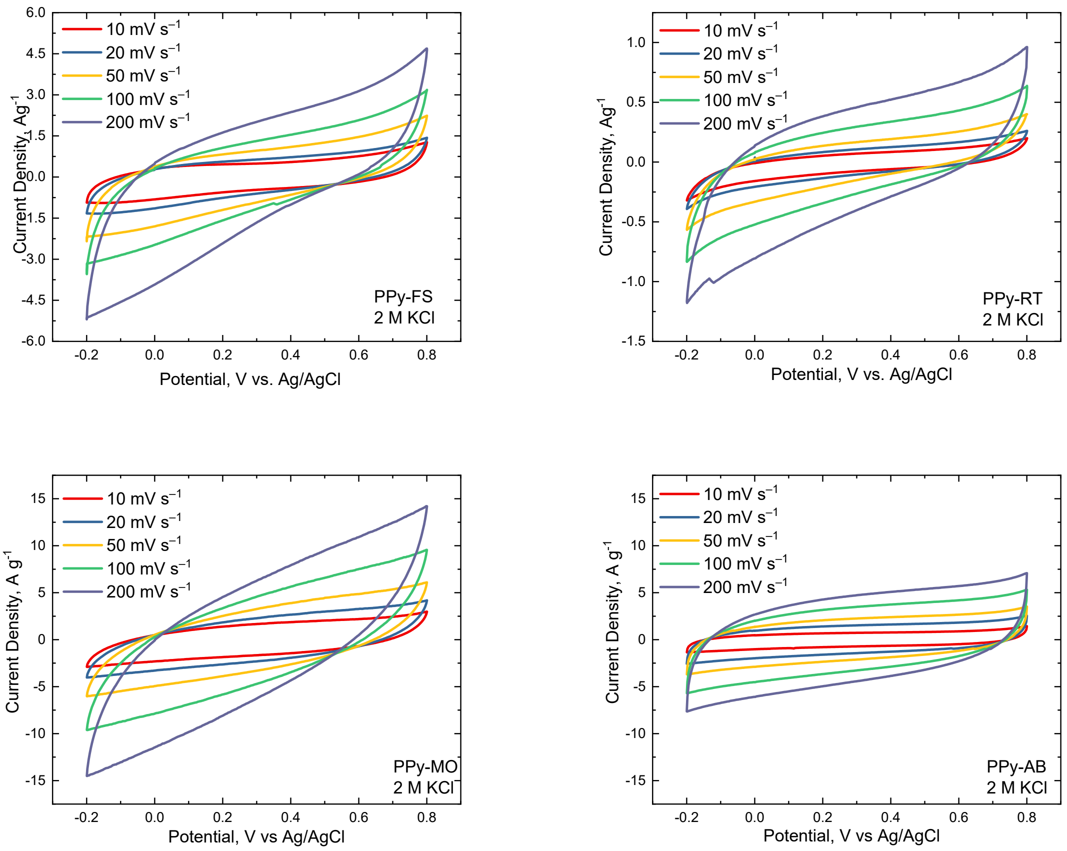

Figure S4. CV curves of PPy-FS, PPy-RT, PPy-AB and PPy-MO measured in 2 M KCl solution at varying scan rates: 10, 20, 50, 100 and 200 mV s^{-1}.

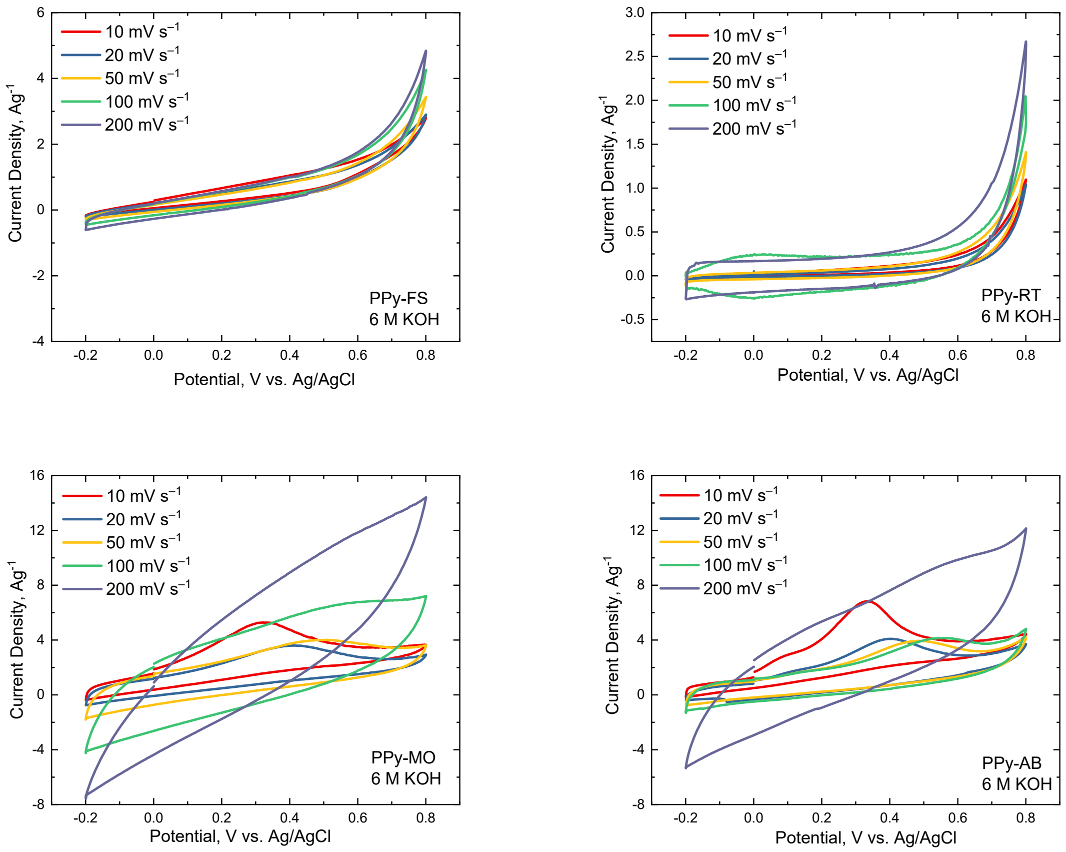

Figure S5. CV curves of PPy-FS, PPy-RT, PPy-AB and PPy-MO measured in 6 M KOH solution at varying scan rates: 10, 20, 50, 100 and 200 mV s^{-1}.

3. Galvanostatic Charge – Discharge Plots

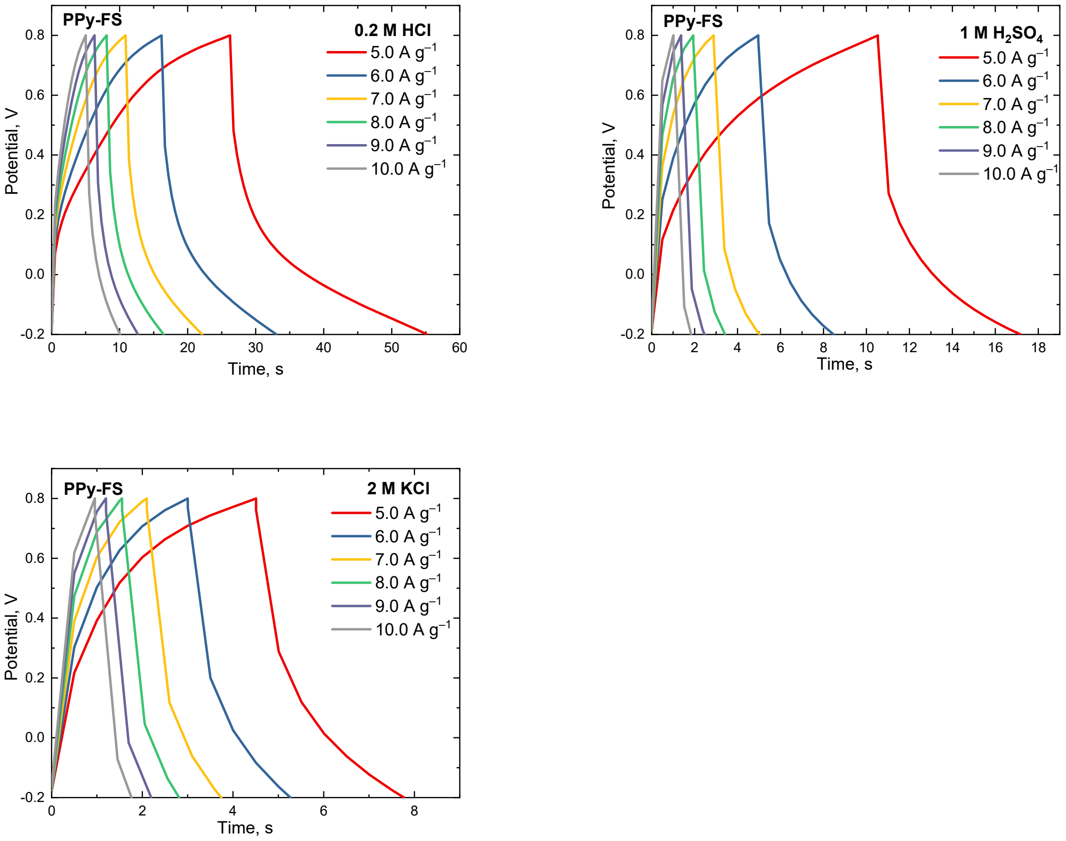

Figure S6. GCD curves of PPy-FS measured in 0.2 M HCl, 1 M H_2SO_4 and 2 M KCl solution at varying applied current density, J: 5.0, 6.0, 7.0, 8.0, 9.0 and 10.0 A g^{-1}.

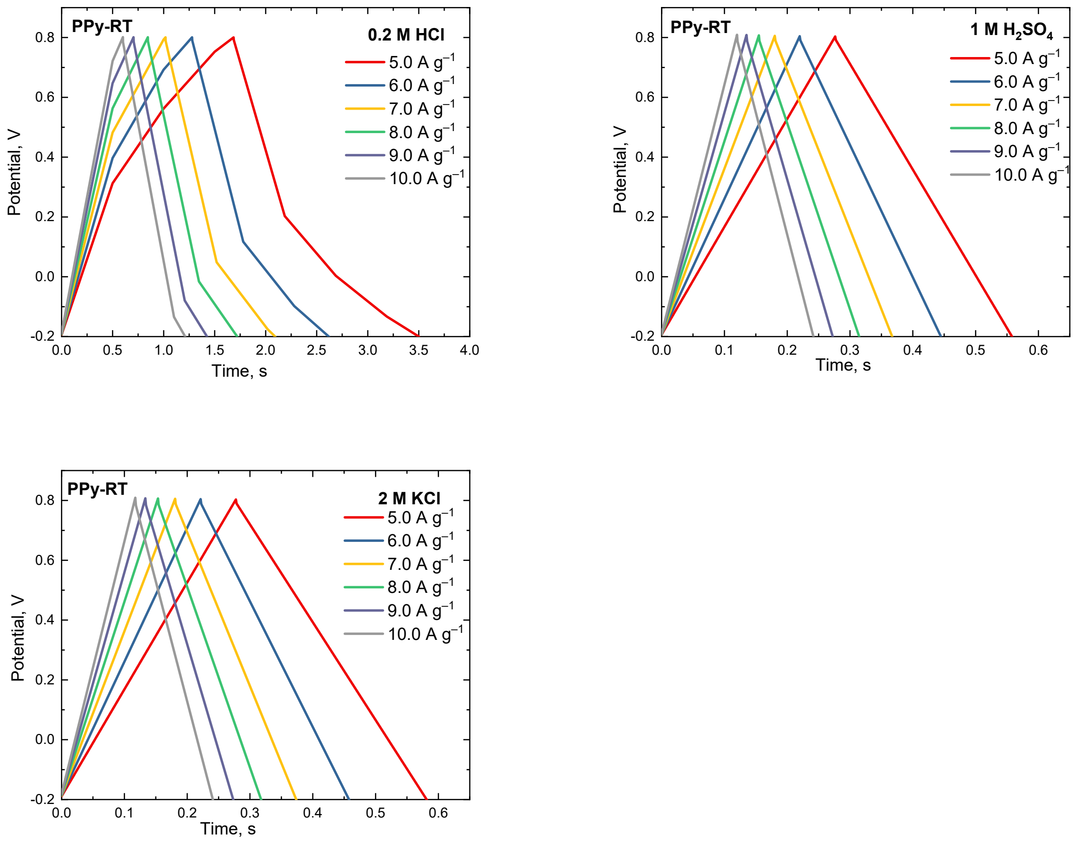

Figure S7. GCD curves of PPy-RT measured in 0.2 M HCl, 1 M H_2SO_4 and 2 M KCl solution at varying applied current density, J: 5.0, 6.0, 7.0, 8.0, 9.0 and 10.0 A g^{-1}.

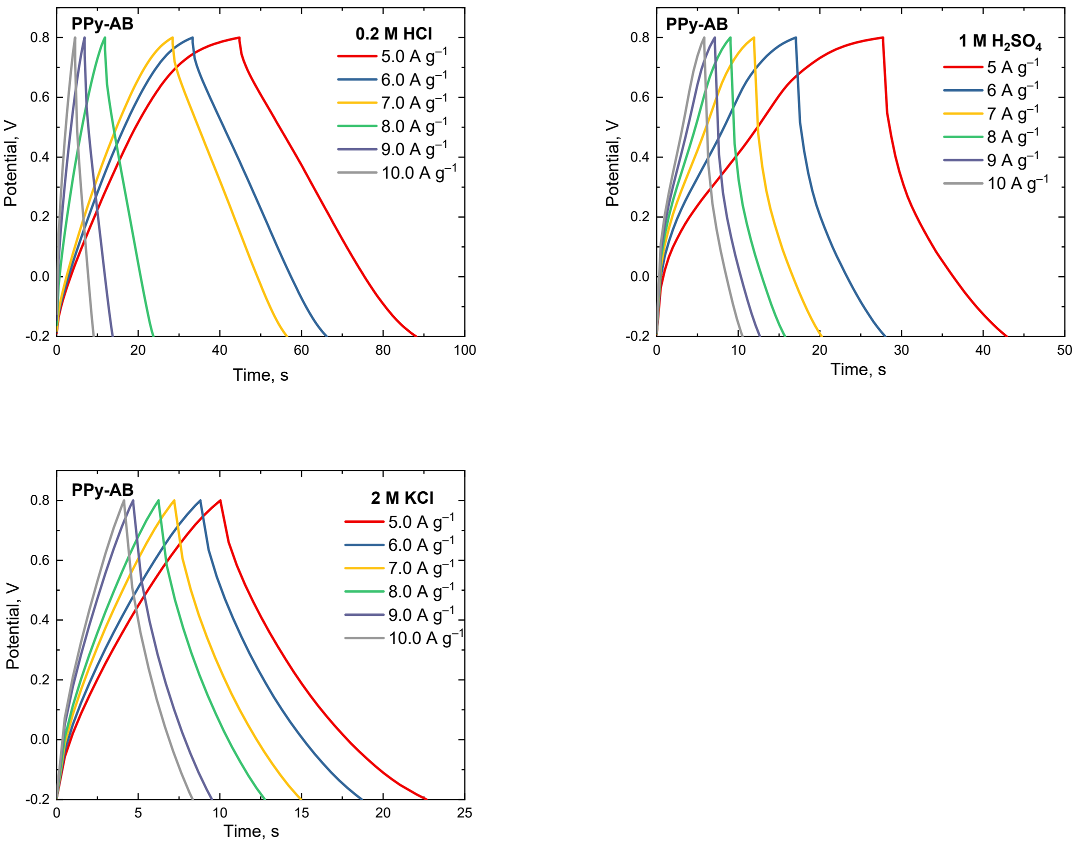

Figure S8. GCD curves of PPy-AB measured in 0.2 M HCl, 1 M H_2SO_4 and 2 M KCl solution at varying applied current density, J: 5.0, 6.0, 7.0, 8.0, 9.0 and 10.0 A g^{-1}.

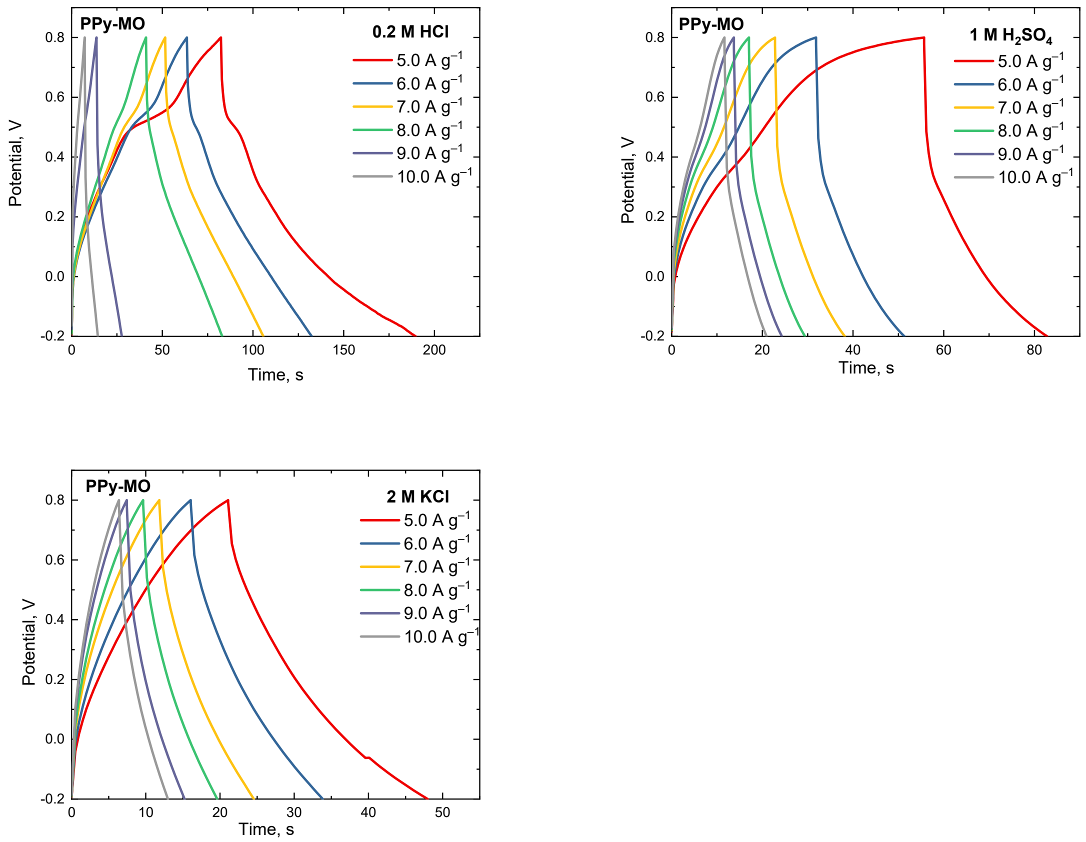

Figure S9. GCD curves of PPy-MO measured in 0.2 M HCl, 1 M H_2SO_4 and 2 M KCl solution at varying applied current density, J: 5.0, 6.0, 7.0, 8.0, 9.0 and 10.0 A g^{-1}.

4. Electrochemical Impedance Spectroscopy and Equivalent Circuit

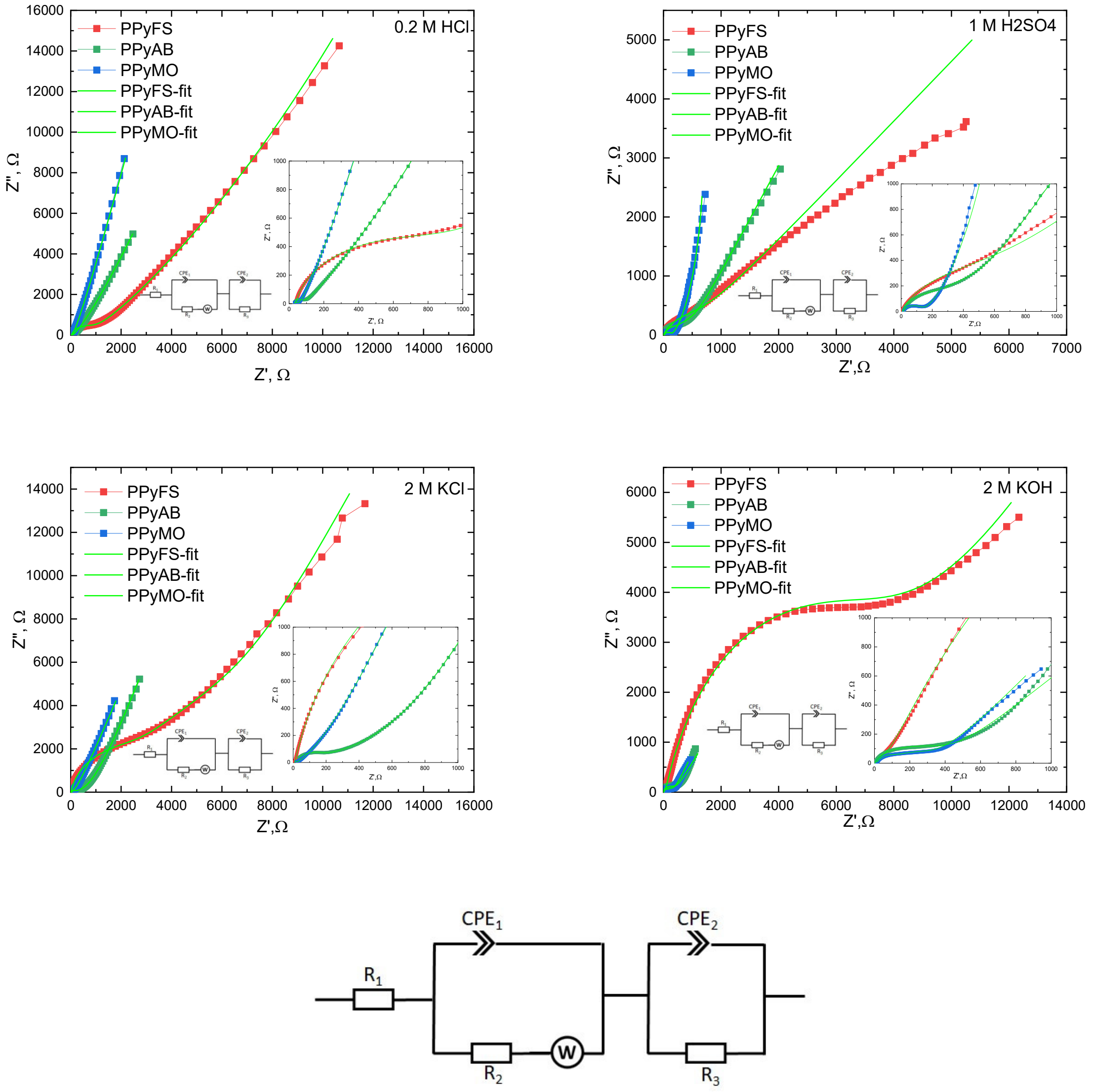

Figure S10. Impedance spectra of PPy and PPy with dyes prepared in frozen-state in 0.2 M HCl, 2M KCl, 6 M KOH, and 1 M H2SO4 and its corresponding equivalent circuit.

5. Calculated Capacitances and estimated resistances in 3-electrode system

Table S1. Computed capacitances of PPy-RT, PPy-FS, PPy-MO and PPy-AB in different aqueous solutions (0.2 M HCl, 1 M H_2SO_4, 2 M KCl and 6 M KOH) based from the CV curves obtained at different scan rates (10, 20, 50, 100 and 200 mV s^{-1}).

	Scan Rate, mV s^{-1}	C, F g^{-1}			
		PPy-RT	PPy-FS	PPy-MO	PPy-AB
HCl	10	20	193	1914	1186
	20	15	119	1373	932
	50	12	68	672	490
	100	10	46	402	323
	200	8.2	32	254	255
KCl	Scan Rate, mV s^{-1}	C, F g^{-1}			
		PPy-RT	PPy-FS	PPy-MO	PPy-AB
	10	6.8	47	136	72
	20	4.7	29	94	71
	50	2.8	17	53	41
	100	2.5	11	38	32
	200	1.9	8.5	26	21
KOH	Scan Rate, mV s^{-1}	C, F g^{-1}			
		PPy-RT	PPy-FS	PPy-MO	PPy-AB
	10	3.6	17	92	98
	20	1.6	7.8	38	44
	50	1.7	3.4	23	18
	100	1.2	2.4	23	10
	200	1.0	1.5	15	14
H_2SO_4	Scan Rate, mV s^{-1}	C, F g^{-1}			
		PPy-RT	PPy-FS	PPy-MO	PPy-AB
	10	9.3	102	169	161
	20	6.5	47	158	107
	50	4.1	20	128	76
	100	3.1	11	106	58
	200	2.1	6.1	81	43

Table S2. Computed capacitances of PPy-RT, PPy-FS, PPy-MO and PPy-AB in different aqueous solutions (0.2 M HCl, 1 M H_2SO_4 and 2 M KCl) based from the GCD curves obtained at different applied current density, J (5.0, 6.0, 7.0, 8.0, 9.0 and 10.0 A g^{-1}).

**HCl**	**J, A g^{-1}**	**C, F g^{-1}**			
		**PPy-RT**	**PPy-FS**	**PPy-MO**	**PPy-AB**
	**5.0**	9.1	145	537	217
	**6.0**	8.0	101	413	197
	**7.0**	7.5	79	379	196
	**8.0**	7.0	67	336	95
	**9.0**	6.5	58	125	62
	**10.0**	6.1	51	73	45
**KCl**	**J, A g^{-1}**	**C, F g^{-1}**			
		**PPy-RT**	**PPy-FS**	**PPy-MO**	**PPy-AB**
	**5.0**	1.5	16	134	63
	**6.0**	1.4	14	107	59
	**7.0**	1.3	12	89	54
	**8.0**	1.3	10	80	52
	**9.0**	1.3	9.0	70	43
	**10.0**	1.2	8.0	66	42
**H_2SO_4**	**J, A g^{-1}**	**C, F g^{-1}**			
		**PPy-RT**	**PPy-FS**	**PPy-MO**	**PPy-AB**
	**5.0**	1.4	33	136	76
	**6.0**	1.3	21	117	66
	**7.0**	1.3	15	108	58
	**8.0**	1.3	12	99	54
	**9.0**	1.2	10	95	50
	**10.0**	1.2	8.2	93	47

Table S3. R_s and R_{ct} values based from the physical interpretation of the EIS spectra of PPy-FS, PPy-MO and PPy-AB in 0.2 M HCl, 1 M H_2SO_4, 2 M KCl and 6 M KOH aqueous electrolytes measured in three-electrode system. (Note: R_s and R_{ct} represent the total resistance and charge-transfer resistance of the electrode, respectively.)

**Resistance, Ω**	**HCl**		
	**PPy-FS**	**PPy-MO**	**PPy-AB**
**R_s**	38.52	31.96	31.35
**R_{ct}**	536.28	20.68	57.98
	**H_2SO_4**		
	**PPy-FS**	**PPy-MO**	**PPy-AB**
**R_s**	5.52	15.45	15.97
**R_{ct}**	631.11	143.12	373.82
	**KCl**		
	**PPy-FS**	**PPy-MO**	**PPy-AB**
**R_s**	8.81	18.28	34.64
**R_{ct}**	3461.71	164.94	186.2
	**KOH**		
	**PPy-FS**	**PPy-MO**	**PPy-AB**
**R_s**	6.59	15.66	5.45
**R_{ct}**	8136.62	371.88	470.43

Table S4. Estimated fitting parameters based on the fitted equivalent circuit. (Note: R_1 and R_2 are the equivalent resistances representing R_s and R_{ct} from Table S3.; EC-Lab software for Nyquist plot fitting was used.)

**Fitting Parameters**	**HCl electrolyte**			**H_2SO_4 electrolyte**		
	**PPy-FS**	**PPy-MO**	**PPy-AB**	**PPy-FS**	**PPy-MO**	**PPy-AB**
**R_1**	34.46	31.9	32.4	5.41	15.1	15.3
**R_2**	438.1	23.4	48.6	687.6	110	255.4
**R_3**	637	5651	1859	295.7	29.748	152.8
**CPE_1**	8.07E-06	3.28E-04	4.65E-07	1.21E-05	7.21E-06	2.09E-04
**CPE_2**	8.95E-07	4.06E-04	2.73E-08	1.16E-03	9.24E-04	1.89E-05
**S**	19.98	9.02	12.53	40.6	499	106
**χ^2**	0.05	0.05	0.05	0.5	0.07	0.05
	**KCl electrolyte**			**KOH electrolyte**		

Fitting Parameters	PPy-FS	PPy-MO	PPy-AB	PPy-FS	PPy-MO	PPy-AB
**R_1**	8.71	18.6	35.6	6.48	15.23	5.71
**R_2**	2830	266.5	117.9	7480	321.9	562.4
**R_3**	2417	82.82	99.07	33.76	58.89	93.64
**CPE_1**	8.23E-05	2.96E-04	3.75E-04	1.28E-05	1.38E-04	5.89E-05
**CPE_2**	1.02E-05	4.94E-07	5.39E-05	4.26E-06	1.82E-06	9.77E-06
**S**	5.87	31.81	15.75	27.7	41.54	630.5
**χ^2**	0.04	0.04	0.03	0.1	0.1	0.1

Table S5. Comparison in capacitance retention and ESR values of the assembled PPy-MO-based supercapacitor to other PPy-based supercapacitors.

Material	ESR, Ω	Capacitance Retention, %	No. of Cycles	Reference
**PPy-MO based supercapacitor**	1.63	89	10000	This Work
**PPy/RGO**	---	83	4000	[1]
**PPy/GO/MnOx**	1.45	96.58	1000	[2]
**PPy-MO-2h**	68.04	71	500	[3]
**PPy@a-NCFs**	0.7	93	5000	[4]
**PPy Hydrogel**	---	90	3000	[5]
**GO-PPy-Ag**	---	93	5000	[6]
**HHAc/PPy**	1.23	79	10000	[7]
**TP2(PPy Hydrogel)**	2.2	90	1000	[8]
**PVA-GO-PPy**	---	97.4	2000	[9]

References

[1] Zhu, J.; Xu, Y.; Wang, J.; Wang, J.; Bai, Y.; Du, X. Morphology controllable nano-sheet polypyrrole–graphene composites for high-rate supercapacitor. Phys. Chem. Chem. Phys. 2015 17 19885-19894. DOI: 10.1039/C5CP02710A

[2] Ng, C.H.; Lim, H. N.; Lim, Y. S.; Chee, W. K.; Huang, N. M. Fabrication of flexible polypyrrole/graphene oxide/manganese oxide supercapacitor. Int. J. Energy Res. 2015 39 344-355. https://doi.org/10.1002/er.3247

[3] Xu, J.; Wang, D.; Fan, L.; Yuan, Y.; Wei, W.; Liu, R.; Gu, S.; Xu, W. Fabric electrodes coated with polypyrrole nanorods for flexible supercapacitor application prepared via a reactive self-degraded template. Org. Electron. 2015 26 292-299. https://doi.org/10.1016/j.orgel.2015.07.054

[4] Wang, Z.; Carlsson, D.O.; Tammela, P.; Hua, K.; Zhang, P.; Nyholm, L.; Strømme, M.; Surface Modified Nanocellulose Fibers Yield Conducting Polymer-Based Flexible Supercapacitors with Enhanced Capacitances. ACS Nano 2015 9 7563–7571. https://doi.org/10.1021/acsnano.5b02846

[5] Bo, J.; Luo, X.; Huang, H.; Li, L.; Lai, W.; Yu, X.Morphology-controlled fabrication of polypyrrole hydrogel for solid-state supercapacitor. J Power Sources 2018 407 105-111. https://doi.org/10.1016/j.jpowsour.2018.10.064

[6] Singu, B.S.; Yoon, K.R. Highly exfoliated GO-PPy-Ag ternary nanocomposite for electrochemical supercapacitor. Electrochim. Acta 2018 268 304-315. https://doi.org/10.1016/j.electacta.2018.02.076

[7] Dubey, P.; Maheshwari, P.H.; Sundriyal, S. Human Hair-Derived Porous Activated Carbon as an Efficient Matrix for Conductive Polypyrrole for Hybrid Supercapacitors. Energy Fuels 2022 36 13218–13228. https://doi.org/10.1021/acs.energyfuels.2c01926

[8] Wang, F.; Du, H.; Liu, Y.; Huang, H.; Yu, X.; Zhu, X.; Li, L. Elastic polypyrrole hydrogels reinforced by TEMPO-oxidized cellulose for supercapacitors. Synth. Met. 2021 282 116952. https://doi.org/10.1016/j.synthmet.2021.116952

[9] Wei, D.; Zhu, J.; Luo, L.; Huang, H.; Li, L.; Yu, X. Fabrication of poly(vinyl alcohol)–graphene oxide–polypyrrole composite hydrogel for elastic supercapacitors. J Mater Sci 2020 55 11779–11791. https://doi.org/10.1007/s10853-020-04833-x
